# Domestic risk factors for increased rodent abundance in a Lassa fever endemic region of rural Upper Guinea

**DOI:** 10.1038/s41598-021-00113-z

**Published:** 2021-10-19

**Authors:** Julia Clark, Laith Yakob, Moussa Douno, Joseph Lamine, N.’Faly Magassouba, Elisabeth Fichet-Calvet, Almudena Mari-Saez

**Affiliations:** 1 London School of Hygiene and Tropical Medicine, London, UK; 2 Projet des Fièvres Hémorragiques en Guinée, Laboratoire de Recherche en Virologie, Conakry, Guinea; 3 Mercy Hospital Research Laboratory, Bo, Sierra Leone; 4 Department of Virology, Bernard Nocht Institute of Tropical Medicine, Hamburg, Germany; 5 Centre for International Health Protection, Robert Koch Institute, Berlin, Germany

**Keywords:** Risk factors, Animal behaviour

## Abstract

Lassa fever (LF) is a viral haemorrhagic fever endemic in West Africa and spread primarily by the multimammate rat, *Mastomys natalensis*. As there is no vaccine, reduction of rodent-human transmission is essential for disease control. As the household is thought to be a key site of transmission, understanding domestic risk factors for *M. natalensis* abundance is crucial. Rodent captures in conjunction with domestic surveys were carried out in 6 villages in an area of rural Upper Guinea with high LF endemicity. 120 rodent traps were set in rooms along a transect in each village for three nights, and the survey was administered in each household on the transects. This study was able to detect several domestic risk factors for increased rodent abundance in rural Upper Guinea. Regression analysis demonstrated that having > 8 holes (RR = 1.8 [1.0004–3.2, p = 0.048), the presence of rodent burrows (RR = 2.3 [1.6–3.23, p = 0.000003), and being in a multi-room square building (RR = 2.0 [1.3–2.9], p = 0.001) were associated with increased rodent abundance. The most addressable of these may be rodent burrows, as burrow patching is a relatively simple process that may reduce rodent entry. Further study is warranted to explicitly link domestic rodent abundance to LF risk, to better characterize domestic risk factors, and to evaluate how household rodent-proofing interventions could contribute to LF control.

## Introduction

Lassa fever (LF) is a potentially fatal rodent-borne viral haemorrhagic fever (VHF) caused by the Lassa virus (LASV), a virus of the *Arenaviridae* family^1^. LF was first recognized in Lassa, Nigeria in 1969^2,3^, and is endemic in West Africa, primarily in Nigeria and the countries of the Mano River region: Guinea, Sierra Leone, Liberia^4^. There are approximately 200,000–300,000 cases annually, resulting in 5,000–10,000 deaths^5^, though this is likely to be an underestimate. LASV is ubiquitous in some settings, with serosurveys showing greater than 50% seropositivity in sub-regions of Sierra Leone^6^ and Guinea^7^. Generally, the significance of the burden of disease in endemic areas has been underappreciated, and its dynamics have not been well characterized^8^.

It is estimated that up to 80% of cases of LF are mild or asymptomatic^9^. In the 20% of cases with symptoms, they are often non-specific, making the disease difficult to diagnose in areas where other febrile diseases, such as malaria, are also endemic^10^. In severe cases, LF can cause haemorrhage, shock, organ failure and death. The overall case fatality rate is ~ 1–2%, though this can approach 70% in severe hospitalized cases^1^. There is no vaccine, and while treatment with the broad spectrum antiviral ribavirin can improve outcomes if given early in the course of disease, treatment is otherwise limited to supportive measures^11^.

The mainstay of LF control is limiting human exposure. This is done through health education and rodent control to prevent primary rodent-human transmission, and use of infection prevention and control practices to avoid secondary transmission in healthcare settings^12^. With such a limited range of control options, it is critical that their use be as efficient and effective as possible. This requires development of a detailed understanding of risk factors for LF, including characterization of the circumstances in which rodent-human transmission is most likely to occur.

*M. natalensis* was identified as the primary reservoir host of LASV in Sierra Leone in 1972^13^. It is ubiquitous in sub-Saharan Africa and lives semi-commensally with humans in homes and surrounding vegetation^14^. A study in a high LF endemicity area of Upper Guinea found that *M. natalensis* comprised 95–98% of rodents captured in houses^15^. The main route of transmission is thought to be from rodents to humans via direct or indirect contact—including surfaces and foodstuffs—with rodent urine, blood, or saliva^5^. There is further laboratory evidence that LASV can be aerosolized and transmitted via the airborne route^16^. The proportion of *M. natalensis* infected with LASV in Guinea was found to be 11.3%, though this varied from 0% in low endemic zones to up to 32.1% in high endemic zones^17^.

Detailed study of *M. natalensis* population dynamics in rural Guinea by Fichet-Calvet, et al. has revealed that, in high-endemicity rural areas, rodents are mainly concentrated in homes and sites of proximal cultivation, with relative abundance and seroprevalence varying seasonally^14^. Particularly during the dry season (November–April), trapping success was nearly 40 times higher in homes than in proximal cultivations (TS = 17.4 vs TS = 0.45). It is thought that this effect is driven by human agricultural behaviour, with the lighting of bush fires and indoor storage of crops at the end of the rainy season destroying *M. natalensis* habitats in fields and attracting them to homes. Conversely, LASV positivity in rodents was found to be 2–3 times higher in the rainy season, possibly due to improved viral persistence in cooler, wetter conditions. Incidence in humans is reportedly highest in the dry season^18^, suggesting that is when the majority of infective contact between humans and rodents occurs. This further implicates contact with rodents in the household as a key mode of transmission.

Given increased LF incidence in the dry season when rodents are found indoors, a link between domestic risk factors and LF has been posited. Human exposure to *M. natalensis* in households in LF endemic areas can be frequent, with Bonwitt, et al. finding that respondents in Sierra Leone frequently reported having rodents in their homes (92.4%), having contact with rodents (34.2%) and their fluids (52.8%), and that household construction materials were highly suitable for rodent nesting^19^. A case–control study by Bonner, et al. in refugee camps in Sierra Leone found that recent LF cases had elevated odds of living in homes with rodent burrows or with poor external hygiene, with the presence of rodent burrows being in turn associated with poor housing quality^20^. Other work has been more equivocal, with a study in Nigeria finding no significant difference in housing quality between LASV positive and negative individuals, though they did find a positive association between poor hygiene and self-reported LF case status ^21^. The aforementioned study by Kernéis, et al., also found no association between LASV seroprevalence and the presence of rodents in the home in Guinea^22^. Given the mixed nature of these results, further study is needed to understand the role domestic risk factors may play in the dynamics of LASV. Since LF control relies heavily on rodent control efforts, disentangling the potential associations between household level characteristics and rodent abundance is vital to ensure that efforts can be appropriately targeted.

The main aim of this study is to characterize the nature of domestic spaces in high LF endemicity villages in Faranah, Guinea to determine if features of those spaces are associated with increased abundance of *M. natalensis* rodents. Previous studies have indicated that household construction and the presence of burrows may be associated with LF risk, and, it is thought that indoor storage of food may be a key reason that *M. natalensis* are attracted to homes. Therefore, this study investigates if household porosity—signified by the total number of holes in a room and the presence of rodent burrows—is associated with increased rodent abundance, and if indoor food storage is associated with increased rodent abundance.

## Results

### Demographic characteristics and rodent occurrence

The study included 405 participants who provided information on an estimated 448 unique rooms in 377 unique buildings (Table S1). Participants were mostly adults aged 25–49, approximately 71% were female, almost 60% were Malinke, two thirds had no formal education, and the majority worked either in agriculture or in the home. Accounting for multiple sampling of rooms over the three trapping sessions, the survey was administered and trapping conducted in 585 rooms. Contact with rodents was common, with more than 70% of participants reporting both contact with rodents themselves and with rodent excrement. Pet ownership was relatively uncommon, with 11% of participants owning a cat, and approximately 16% owning a dog. There were 13 rooms where the survey, trapping, or both were not completed due to refusal of consent or absence of the resident. These are excluded from the analysis.

**Figure 1:**
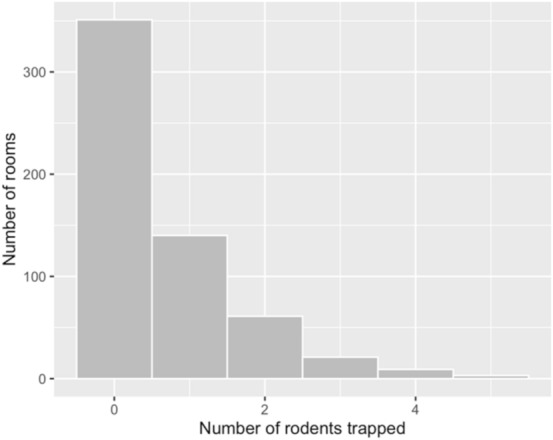
Histogram of rodent captures by room.

Across the 585 rooms, there were a total of 376 rodent captures over the 3,600 trapping nights, with at least one capture occurring in 234 (40.0%) rooms. A histogram showing the distribution of the number of rodent captures is shown in Fig. 1. Overall, the mean number of captures was 0.64 per room (95% CI 0.56–0.72, SD: 0.97), with a minimum of zero and a maximum of five. The variance of rodent abundance was 0.94, which was higher than the mean, indicating the data may be over dispersed. Village was also associated with rodent abundance, with Dalafilani having a lower rate of captures (RR = 0.57 [0.36–0.88], p = 0.008, Table 1).

**Table 1:** Distribution of domestic variables, including crude incident RRs for rodent abundance (n = 585).

	Number (%) of rooms	Number (%) of rodent captures	Crude RR (95% C.I.)	P-value (LRT)
**Village**				
Brissa	120 (20.5)	76 (20.2)	1.0	0.008
Damania	115 (19.7)	87 (23.1)	1.2 (0.82–1.7)	
Sonkonyah	120 (20.5)	90 (23.9)	1.2 (0.82–1.7)	
Dalafilani	114 (19.5)	41 (10.9)	0.57 (0.36–0.88)	
Sokourala	57 (9.7)	43 (11.4)	1.19 (0.75–1.9)	
Yerewalia	59 (10.1)	39 (10.4)	1.0 (0.65–1.7)	
**Building type**				
Round single room	384 (65.6)	226 (60.1)	1.0	0.07
Multiroom square	201 (34.4)	150 (39.9)	1.3 (0.98–1.6)	
**Floor material**				
Cowdung/clay	421 (72.0)	266 (70.7)	1.0	0.7
Cement/other	164 (28.0)	110 (29.3)	1.1 (0.8–1.4)	
**Floor condition**				
Polished	235 (40.2)	137 (36.4)	1.0	0.5
Partly damaged	247 (42.2)	168 (44.7)	1.2 (0.88–1.5)	
Damaged	103 (17.6)	71 (18.9)	1.2 (0.83–1.7)	
**Room purpose**				
Sleeping room	405 (69.2)	262 (69.7)	1.0	0.7
Kitchen	94 (16.1)	95 (25.2)	1.0 (0.73–1.5)	
Store	74 (12.6)	75 (19.9)	0.98 (0.67–1.4)	
Parlour	12 (2.1)	4 (1.1)	0.52 (0.15–1.4	
**Porosity level**				
Low (0–2 holes)	101 (17.3)	41 (10.9)	1.0	0.000002
Medium (3–5 holes)	279 (47.7)	153 (40.7)	1.4 (0.92–2.0)	
High (6–8 holes)	156 (26.7)	116 (30.9)	1.8 (1.2–2.8)	
Very high (8 + holes)	49 (8.4)	66 (17.6)	3.3 (2.1–5.4)	
**Burrows present**				
No	235 (40.2)	88 (23.4)	1.0	0.000000008
Yes	350 (59.8)	288 (76.6)	2.2 (1.7–2.9)	
**Food present**				
No	72 (12.3)	34 (9.0)	1.0	0.1
Yes	513 (87.7)	342 (91.0)	1.4 (0.94–2.2)	
**Water present**				
No	141 (24.1)	76 (20.2)	1.0	0.1
Yes	444 (75.9)	300 (79.8)	1.3 (0.93–1.7)	
**Garbage storage location**				
Far from the house	155 (26.5)	71 (18.9)	1.0	0.04
Outside	396 (67.7)	278 (73.9)	1.5 (1.1–2.1)	
Inside	5 (0.9)	5 (1.3)	2.2 (0.6–7.2)	
Other	29 (5.0)	22 (5.9)	1.7 (0.91–3.0)	
**Exterior grass present**				
No	228 (39.0)	129 (34.3)	1.0	0.1
Yes	357 (61.0)	247 (69.2)	1.2 (0.94–1.6)	
**Season**				
Rainy	237 (40.5)	148 (39.4)	1.0	0.7
Dry	348 (59.5)	228 (60.6)	1.0 (0.81–1.4)	

### Domestic variables and rodent abundance: univariate analysis

We analysed the distribution of the key domestic level variables measured by the survey, including crude incident rate ratios for rodent abundance (Table 1). Buildings in the village are generally either: (1) round single-room structures made from cow-dung plastered mud bricks with thatched roofs, or (2) rectangular, multi-room buildings made from baked and/or cement bricks with tin roofs. Households either consist of a single standalone building or multiple buildings located near each other. Approximately two thirds (65.5%) of rooms were located in round single room buildings. When examining univariate associations between domestic risk factors and rodent abundance, there was some evidence that rodent abundance varied by building type with the rate of rodent capture being higher in rooms located in multiroom square buildings compared to round single room buildings (RR = 1.3 [0.98–1.6], p = 0.07, Table 1).

Majority of floors are made from cow dung or clay and some women repair them often, especially before the harvest, 72.0% of floors were made from cow dung or clay and 40.2% were polished. Most rooms were used for sleeping (69.2%), though kitchens (16.1%) and storerooms (12.6%) were also common. None of these variables, floor material, floor conditions and room purpose was significantly associated with rodent capture (Table 1).

More than three quarters of rooms had at least three holes, and rodent burrows specifically were present in 60% of rooms (Table 1). At the room level, porosity level and presence of burrows appeared to be associated with increased rates of rodent capture. Negative binomial regression analysis indicated that a room having 8 + holes was associated with a greater than three-fold increase in the rate of rodent capture (RR = 3.3 [2.1–5.4], p < 0.001), the presence of burrows was associated with 2.2 times the rate of rodent capture (RR = 2.2 [1.7–2.9], p < 0.001, Table 1). Food is generally stored inside, in plastic bags, containers with and without lid or directly on the floor, though some households have designated store rooms either in the house or in a nearby building. Food was stored in a large majority of rooms (87.7%). Water is also commonly stored inside, often in plastic containers or jars that may be covered or uncovered***.*** Surprisingly, the presence of food inside was not significantly associated with rodent captures (RR = 1.4 [0.94–2.2], p = 0.1, Table 1). Only garbage stored outside the house was associated with rodent captures (RR = 1.5 [1.1–2.1], p = 0.04, Table 1). Presence of grass outside the building and season had no effect on rodent captures (Table 1).

### Domestic variables and rodent abundance: multivariate analysis

To more fully characterize domestic variables associated with rodent abundance, a multiple regression model was constructed. Table 2 shows the main regression results for key variables at the main stages in the model fitting process. Initially, generalized linear models including rodent abundance and the main exposures were fit with both poisson and negative binomial distributions. When the poisson and negative binomial models were compared using the likelihood ratio test, the negative binomial model was superior (p < 0.001, Table 2) and, as such, was used as the underlying distribution for all further model fitting.

**Table 2:** Regression results for rodent abundance for crude and adjusted multiple regression models (n = 585). Results are shown as regression coefficients for key variables, with standard errors reported in parentheses, and the overall model log likelihood.

	Crude poisson	Crude negative binomial	Adjusted negative binomial–random effects	Adjusted negative binomial–random effects + a priori variables	Fully adjusted negative binomial
Constant	− 1.2 (0.22)	− 1.2 (0.25)	− 1.47 (0.25)	− 1.81 (0.32)	− 2.4 (0.41)
> 8 holes	0.58 (0.24)**	0.58 (0.28)**	0.45 (0.28)	0.54 (0.30)*	0.58 (0.30)*
Presence of burrows	0.66 (0.16)***	0.66 (0.18) ***	0.73 (0.18)***	0.85 (0.18) ***	0.84 (0.18)***
Presence of food	0.28 (0.18)	0.28 (0.21)	0.32 (0.20)*	0.31 (0.20)	0.29 (0.20)
Building type	−	−	−	0.57 (0.20)***	0.67 (0.21)***
Log Likelihood	− 631.9	− 619.8	− 612.7	− 597.7	− 592.9

*, **, *** indicates significance at the 90%, 95%, and 99% level respectively.

This model was then refit as a generalized linear mixed model to assess the potential random effects of building, and room. Likelihood ratio tests comparing to a model with no random effects revealed that a model with a random effect at the building and room levels was superior to a model without random effects (p < 0.001, Table 2). After fitting the random effects, additional variables were added to adjust for potential confounding and incorporate additional exposures of secondary interest. The first of these were the a priori confounders were village, season, building type, and floor material. After fitting this model, the variance inflation factor was not greater than three for any variable and they were all retained. Additional potential confounders were added, such that the final adjusted model included the variables rodent abundance, number of holes, burrow presence, food presence, water presence, presence of grass in the surrounding area, garbage location, floor condition, village, season, building type, rooms purpose, and floor material. This resulted in a model with a total of 23 parameters, which is appropriate for a model with n = 585. The overall p-value for the fully adjusted model, compared to a null model, was < 0.001 (Table 2). A table with the variance inflation factors for each variable in the fully adjusted model is included in the supplementary information (Table S2).

**Figure 2:**
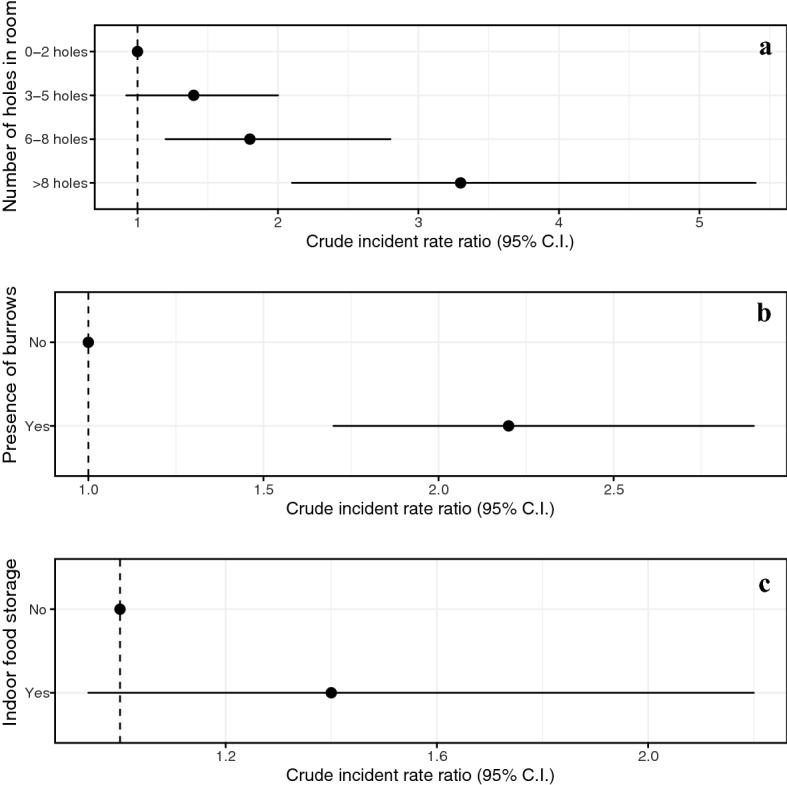
Forest plots showing adjusted incident RRs for room-level rodent abundance by (**a**) number of holes in a room, by (**b**) presence of burrows, and by (**c**) indoor food storage.

Compared to rooms with < 3 holes, the adjusted RRs for porosity were 0.92 (95% CI 0.59–1.54 p = 0.7) for rooms with 3–5 holes, 0.90 for rooms with 5–8 holes (95% CI 0.53–1.5, p = 0.7), and 1.8 for rooms with > 8 holes (95% CI 1.0–3.2, p = 0.048). The adjusted RR was 2.3 for the presence of rodent burrows (95% CI 1.6–3.3, p < 0.001) and 1.3 the presence of food (95% CI 0.90–2.0, p = 0.2). For the exposures of secondary interest, rodent captures were two times more common in multiroom square buildings compared to round single room buildings, with an adjusted RR of 2.0 (95% CI 1.3–2.9, p = 0.001) (Fig. 2, Table S1). Significant relationships such as storing garbage outside the home and Sokourala village associated with increased rodent abundance in the crude model turned to non-significant in the adjusted model (RR = 1.3 [0.96–1.8], p = 0.09 and RR = 1.6 (0.96–2.8), p = 0.07 respectively). There were no other associations with rodent abundance detected in the final adjusted model.

## Discussion

Household characteristics are commonly presented as a driver for rodent presence in the Lassa endemic areas, however limited studies have looked at this correlation and its potential risk^20^. As the majority of studies of *M. natalensis* abundance have focused exclusively on observation of the exterior of the home or dynamics in natural habitats, there has been little study of how factors operating inside the home, e.g., food storage, can be associated with rodent abundance. As such, the associations found in this study are novel. Our study showed that household porosity, the presence of burrows and the building type were the risk factors associated with the rate of rodent capture in the six surveyed villages in the Faranah region of Upper Guinea. Surprisingly, presence of food or garbage inside did not appear as a risk factor for presence of rodents.

### Household porosity

Two of the main exposures which referred to construction porosity—the number of holes, and the presence of burrows—were associated more strongly than would be expected by chance with an increased number of rodent captures. While there was evidence that having a large number of holes, i.e., more than 8, was highly associated with increased rodent abundance, there was no evidence to support this association for rooms with 3–5 or 5–8 holes, indicating that the rate of rodent capture is not significantly elevated at those levels of porosity. The presence of burrows was a clear indicator of rodent activity, and even more so than porosity, indicates a specific attempt to enter a particular room. The presence of rodent burrows have been found to be associated both with LF cases^20^ and *M. natalensis* trapping success in Sierra Leone^23^, with the latter also found here. The association between burrow presence and rodent abundance also extends findings linking the two in natural habitats to domestic spaces, and suggests that the presence of burrows in the home can also be a suitable proxy for rodent abundance.

Interestingly, building type was also strongly associated with rodent abundance, with multi-room square houses having twice the rate of rodent captures as round, single room buildings. This is somewhat surprising, as multi-room square houses are typically constructed of baked or cement bricks, which are more durable than the mud bricks and clay used for single room buildings. This may be partially due to size—multiroom buildings are larger and pose more opportunities for rodent entry, which may make captures more likely. Multiroom houses are considered more secured for storing rice, and one of the rooms can serve as food store for several families. Importantly, this finding implies that simply encouraging construction with more durable materials may not be sufficient if there are still gaps in the construction, including rodent burrows, that allow for rodent entry.

It is plausible that the associations observed between rodent abundance at the room level and the porosity of the room, presence of burrows, and building type are causal. Rooms that are highly porous are more easily accessible to rodents, making the association between the two expected. It is however; more difficult to explain why there is only evidence to support the association when there are more than 8 holes in a given room and not at lower levels of porosity. One potential explanation is that highly porous rooms are more likely to have at least one accessible entry point at all times, making it more likely that a rodent will enter during the limited period of rodent trapping. It is also possible that high porosity is indicative of generally poor construction quality. Infrastructural deficiencies such as un-plastered walls in the home have been found to be associated with both increased probability of rodent entry into houses, and markers of *Leptospira* transmission, another rodent-borne disease^24^.

### Action

High structural poverty levels and geographical remoteness limit the accessibility of durable construction materials, and almost all houses have at least some gaps, even when made from durable materials such as cement. Targeted efforts to address gaps from burrows and those around doors/windows or between the wall and roof may be feasible. Patching burrows, which is currently done in the study area, may be particularly useful, as it has the benefit of directly addressing a specific risk factor for rodent abundance, while also reducing household porosity overall. Burrow patching should, however, be done with durable materials because rodents frequently re-dig burrows^25^. An intervention combining rodent control and actions for reducing household porosity could not only reduce the size of the rodent population but increase the difficulty of recolonization of houses by rodents. To explore the possibility of such an intervention, a pilot study including local participants and their views, stakeholders and private sector will be fundamental.

### Presence of food

The study did not identify an association between presence of food and increased rodent captures. This is somewhat surprising, as it has been posited that the storage of food in houses is a key factor in attracting *M. natalensis* to homes^14^. This can potentially be explained by the times of year in which the study occurred. The June-July study period fell during the rainy season, when food sources outside homes are plentiful. During the November–December study period, food is starting to be stored in houses, but is also available in the rice fields. As such, food may have been relatively abundant both inside and outside the house during the study periods, reducing the degree to which rodents are attracted to only food inside the home. Another explanation might be the storage system and that when the food is stored in sealed bag it most difficult to be access.

### Action

A large majority of individuals store food in their homes, and external dedicated storage facilities are uncommon. Building dedicated facilities that would enable storage of food outside the home would require significant investment and behaviour change, and may not present a high impact opportunity given the lack of evidence for an association between indoor food storage and rodent abundance. More research needs to be done taking in account the seasonality and quantifying the food present in the room. Innovative ideas for food proofing containers should be explored with communities.

### Household environment

The storage of garbage was associated with increased rodent abundance in the crude model. Rooms located in buildings where garbage was stored outside had a rate of rodent captures approximately 30% higher than rooms where garbage was stored far from the house. It is plausible that garbage stored outside homes is attractive to rodents as a food source. At least one study has found that external features are associated with LF cases^20^, though the measure of external hygiene in that study was an overall score rather than a specific accounting of the surroundings. Further exploration of exterior hygiene, rodent abundance, and potential interventions may be warranted, noting, however, that the association was relatively weak because it disappeared in the adjusted model.

## Conclusions

Rodent presence and contact in the home is extremely common in the region, and such exposure may increase the risk of LASV transmission. At the same time, there is little evidence regarding the efficacy of rodent proofing at the household level, and it is unlikely that rodent control alone is sufficient to reduce LF incidence^26^. However, the results from this study are promising, in that they provide evidence suggesting that there are a number of risk factors at the domestic level that are associated with increased rodent abundance that may be possible avenues for improved rodent control.

Overall, the ecological and epidemiological dynamics of LF are complex, and disentangling the contributions of various risk factors remains challenging. This interdisciplinary research demonstrates that detailed study of risk factors for increased contact between humans and zoonotic reservoirs can be valuable for identifying potential targets for control efforts, particularly within domestic spaces that have been understudied and where much transmission is thought to occur. Housing improvement as a method for rodent control requires further exploration and potentially has applications for a variety of rodent-borne disease beyond LF. Further interdisciplinary study to continue exploring LASV transmission at the domestic level is warranted to ensure that prevention of LF is as successful as possible.

## Materials and methods

### Study site

**Figure 3:**
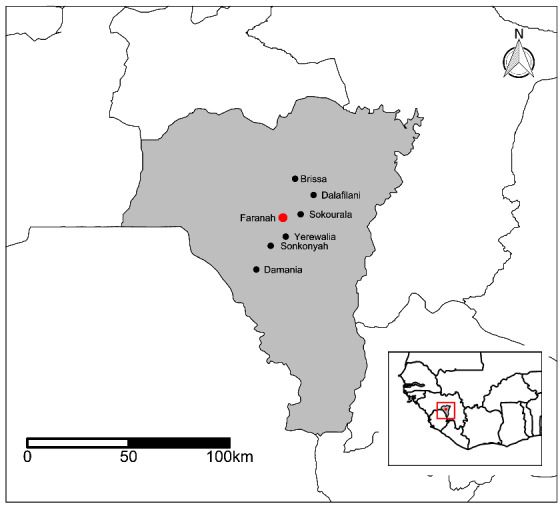
Location of study sites relative to Faranah, Upper Guinea. The map was created in R version 3.5.3 (https://www.r-project.org/). The underlying map of Guinea comes from the Database of Global Administrative Areas (https://gadm.org/index.html).

The study took place in Faranah prefecture in Upper Guinea. The region is located in the Guinean forest-savannah ecoregion^27^ and the study sites themselves were six villages: Brissa, Dalafilani, Damania, Sokourala, Sonkonyah, and Yerewalia (Fig. 3). These villages were selected because the prevalence of LASV in the rodent population is ranging from 7 to 22%, and they are accessible by road, located not more than ~ 60 min by car from Faranah, have population sizes of < 1,000 people. This is large enough to ensure there are sufficient rooms to enable the placement of 120 traps, while also being small enough that *M. natalensis* is the dominant rodent species and unlikely to be displaced by the urban-adapted *Rattus rattus*^28^.

To account for the seasonal dynamics of LASV and *M. natalensis*, there were three fieldwork sessions covering both the dry and rainy seasons. An initial dry season pilot took place in January 2018, and included Dalafilani and Sokourala. A wet season session took place from June-July 2018 and included Brissa, Damania, Sonkonyah, and Dalafilani. A final dry season session took place in November–December 2018 and included Brissa, Damania, Sonkonyah, and Yerewalia. Since sampling occurred in the same village more than once across the three fieldwork sessions, some rooms and/or buildings were sampled on more than one occasion.

### Domestic survey

The domestic survey was administered at the household level and information was gathered on rooms where rodent trapping occurred. The survey itself was designed and tested in two villages in Faranah in January 2018. Survey development was informed by prior anthropological data on domestic spaces in the region. This enabled the development of questions capable of gathering finely grained, context-specific descriptions of domestic spaces. Questions regarding the subject’s personal information covering demographic information (e.g., age, sex, education, occupation, ethnicity), interactions with rodents (e.g., contact with rodent excreta, touching rodents, seeing rodents in the home, use of poison, etc.), and pet ownership were asked directly. Household construction (e.g., building materials, presence of gaps in the roof, doors, and windows, and presence of rodent burrows), household surroundings, food/water storage, the organization of possessions were assessed by observation, in presence of the owner of the room or house. Interviews were conducted in Malinke or French by a native speaker who was familiar with the survey and its objectives. As a result, interviewer bias related to the phrasing of questions was reduced to the extent possible. All data was collected via smartphone using the platform SurveyCTO.

### Rodent trapping

A total of 120 traps were placed along a transect in each village. Two folding Sherman live traps (Sherman Live Trap Co., Tallahassee, FL, USA) baited with a mixture of dried fish, peanuts, and wheat flour were placed in each room, up to a maximum of 12 per household. Each household with traps was marked and its latitude and longitude were recorded. The traps remained in place for three nights, and were checked for captured rodents each morning. Captured rodents were then euthanized and necropsied in the field—at a site a safe distance from the village and under appropriate biosafety protocols—to collect biological samples to a biosafety level 3 procedure^29,30^. Traps that successfully captured rodents were recorded, cleaned, rebaited and replaced in their original locations if further trapping nights remained. After three nights, all traps were collected. There were thus 360 trapping nights per village for a total of 3,600 trapping nights overall across the three fieldwork sessions.

### Ethic statement

Approval for the study was obtained from Guinean National Ethics Committee and the London School of Hygiene and Tropical Medicine (permit n° 129/CNERS/16 and 070/CNERS/18). The methods were carried out in accordance with the approved guidelines. As approved by local ethics committee (Comité National d’Ethique pour la Recherche en Santé) we conducted the trapping of the rodents (see rodent trapping section). The relevant guidelines were followed for the study on rodents and conducted in compliance with ARRIVE guidelines. In all cases, one household participant provided written informed consent, when people couldn’t write a witness provided the written consent, to participate in the study. All participants were provided with a copy of the consent form and an information sheet.

### Data analysis

#### Data preparation

A multiple regression analysis was conducted to identify associations between key features of domestic spaces and rodent abundance. Rodent abundance was defined as the number of trapped rodents per room. In accordance with the hypotheses that room porosity and food storage are associated with increased rodent abundance, the number of holes in a room, the presence of burrows, and indoor food storage were the main exposures of interest. Other variables were considered to be exposures of secondary interest or potential confounders. Data were prepared for analysis in Excel, and analyses were then conducted in R^31^.

During data preparation, each building and room was assigned an identification number. Since some villages were sampled multiple times across the three fieldwork sessions, geolocation and demographic information was used to attempt to identify buildings and rooms that were sampled in more than one trapping session, though Identification may be imperfect as it is challenging to definitively match buildings and rooms across multiple trapping sessions since the path of the transect varies slightly from session to session for logistical reasons (e.g., absence of the resident). Survey data was then linked to room level rodent captures. Due to the large number of variables addressing construction porosity in the survey, an overall porosity score was compiled. This referred to total number of holes detected in household construction, including between the roof and walls, in and around the doors and windows, and from rodent burrowing. Histograms, frequency tables, and univariate regressions were used to explore associations between domestic variables and rodent abundance formally.

#### Models

- *Crude model and main exposure variables*
  To develop the multiple regression model, a determination was made regarding the appropriate underlying probability distribution. Particular attention was paid to the distribution, as overdispersion, i.e., variance greater than the mean, would indicate that a negative binomial distribution may be appropriate rather than a poisson distribution, which would typically be used for non-overdispersed count data^32^. To evaluate this formally, generalized linear models with poisson and negative binomial distributions including rodent abundance and the main exposure variables (number of holes, food presence, and presence of burrows) were fit using the MASS package in R^33^. These initial models were then compared using the likelihood ratio test^34,35^. The superior model under this procedure was considered to be the initial crude model.
- *Adjusted model and secondary exposure variables*
  This crude model was then adjusted to account for clustering, confounding, and to evaluate the role of secondary exposures of interest. Potential clustering was considered at three levels: (1) village, (2) building, and (3) room. Villages may have different underlying rodent ecology, and multiple sampling of the same villages, buildings and/or rooms across trapping sessions results in non-independent observations.

A generalized linear mixed model^36^ with a negative binomial probability distribution, the same variables as the initial model, and a random effect were fit separately for each potential clustering variable using the lme4 package^37^. Each of these models was then compared to a generalized linear model without random effects using the likelihood ratio test to determine which random effects were appropriate. After accounting for clustering, confounding was considered. Inclusion of additional explanatory parameters was favoured so long as the variables were not overly multicollinear and there were at least ten observations per parameter to avoid overfitting^38^.

Model construction proceeded as follows: variables not already included that were thought to be strong a priori confounders—in this case, village, season, building type, and floor material—were added to the model. Multicollinearity was then evaluated via variance inflation factors (VIFs) using the corvif function developed by Zuur^39^. If any variable had a VIF greater than three, the variable with the highest VIF was dropped, the model was refitted, and the VIFs were recalculated^40^. This procedure was repeated until all variable VIFs were less than three^40^. After including all a priori confounders of interest that did not exhibit multicollinearity, variables of interest as secondary exposures and/or additional potential confounders (i.e., variables with strong univariate associations with rodent abundance that were not on the causal pathway) were added. Multicollinearity was then re-evaluated and variables were removed as appropriate.

## Supplementary Information

Supplementary Information 1.

Supplementary Information 2.

## References

[1] Houlihan C, Behrens R (2017). Lassa fever. Br. Med. J. Publ. Group.

[2] Frame JD, Baldwin JM, Gocke DJ, Troup JM (1970). Lassa fever, a new virus disease of man from West Africa. I. Clinical description and pathological findings. Am. J. Trop. Med. Hyg..

[3] Buckley SM (1970). Lassa fever a new virus disease of man from West Africa. III. Isolation and characterization of the virus. Am. J. Trop. Med. Hyg..

[4] Fichet-Calvet E, Rogers DJ (2009). Risk maps of Lassa fever in West Africa. PLoS Negl. Trop. Dis..

[5] JB, M. Lassa Fever. Emergence and Control of Rodent-Borne viral diseases. (1999).

[6] McCormick JB, Webb PA, Krebs JW, Johnson KM, Smith ES (1987). A prospective study of the epidemiology and ecology of Lassa fever. J. Infect. Dis..

[7] Lukashevich IS, Clegg JC, Sidibe K (1993). Lassa virus activity in Guinea: distribution of human antiviral antibody defined using enzyme-linked immunosorbent assay with recombinant antigen. J. Med. Virol..

[8] Gibb R, Moses LM, Redding DW, Jones KE (2017). Understanding the cryptic nature of Lassa fever in West Africa. Pathog. Glob. Health.

[9] Richmond JK, Baglole DJ (2003). Lassa fever: Epidemiology, clinical features, and social consequences. BMJ.

[10] Hamblion EL, Raftery P, Wendland A, Dweh E, Williams GS, George RNC, Soro L, Katawera V, Clement P, Gasasira AN, Musa E, Nagbe TK (2018). The challenges of detecting and responding to a Lassa fever outbreak in an Ebola-affected setting. Int. J. Infect. Dis..

[11] Hadi CM, Khan SH, Bangura J, Sankoh M, Koroma S (2010). Ribavirin for Lassa fever postexposure prophylaxis. *Emergent Infectious Disease*. Centers Dis. Control Prevent..

[12] Senior K (2009). Lassa fever: Current and furture control options. Lancet Infect. Dis..

[13] Monath TP, Newhouse VF, Kemp GE, Setzer HW, Cacciapuoti A (1974). Lassa virus isolation from Mastomys natalensis rodents during an epidemic in Sierra Leone. Science.

[14] Fichet-Calvet E, Lecompte E, Koivogui L, Soropogui B, Dore A, Kourouma F, Sylla O, Daffis S, Koulemou K, Ter Meulen J (2007). Fluctuation of abundance and Lassa virus prevalence in Mastomys natalensis in Guinea, West Africa. Vec. Borne Zoo. Dis..

[15] Fichet-Calvet E, Audenaert L, Barriere P, Verheyen E (2010). Diversity, dynamics and reproduction in a community of small mammals in Upper Guiena, with emphasis on pygmy mice ecology. Afr. J. Ecol..

[16] Stephenson EH, Larson EW, Dominik JW (1984). Effect of environmental factors on aerosol-induced Lassa virus infection. J. Med. Virol..

[17] Lecompte, E. F.-C., E..Daffis, S..Koulemou, K.,Sylla, O.,Kourouma, F.,Dore, A.,Soropogui, B.,Aniskin, V.,Allali, B.,Kouassi Kan, S.,Lalis, A.,Koivogui, L.,Gunther, S.,Denys, C., ter Meulen, J. Mastomys natalensis and Lassa fever, West Africa. *Emerg Infect Dis* **12**, 1971–1974, doi:10.3201/eid1212.060812 (2006). 10.3201/eid1212.060812 PMC3291371 17326956

[18] McCormick JB (1986). Clinical, epidemiologic, and therapeutic aspects of Lassa fever. Med. Microbiol. Immunol..

[19] Bonwitt J, Lamin J, Ansumana R, Dawson M, Buanie J (2017). At home with mastomys and rattus: Human–rodent interactions and potential for primary trnasmission of lassa virus in domeistic spaces. Am. J. Trop. Med. Hyg..

[20] Bonner PC, Belmain SR, Oshin B, Baglole D, Borchert M (2007). Poor housing quality incrases risk of rodent infestation and Lassa Fever in refugee camps of Sierra Leone. Am. J. Trop. Med. Hyg..

[21] Ochei O, Okoh E, Abah SO (2014). Housing factors and transmission of Lassa Fever in a rural area of south-south Nigeria. Gen. Heal. Med. Sci..

[22] Kerneis S, Koivogui L, Magassouba N, Koulemou K, Lewis R, Aplogan A, Grais RF, Guerin PJ, Fichet-Calvet E (2009). Prevalence and risk factors of Lassa seropositivity in inhabitants of the forest region of Guinea: A cross-sectional study. PLoS Negl. Trop. Dis..

[23] Moses, L.M., Kargbo, K., Koninga, J., Veltus, E., Lewinski, J.P., et al. Household predictors of abundance of the Lassa virus reservoir, Mastomys natalensis, in the Eastern Province of Sierra Leone. in 58th annual meeting of the American Society of Tropical Medicine and Hygiene 18–22 (Washington, DC, 2009).

[24] Costa F, Ribeiro GS, Felzemburgh RD, Santos N, Reis RB, Santos AC, Fraga DB, Araujo WN, Santana C, Childs JE, Reis MG, Ko AI (2014). Influence of household rat infestation on leptospira transmission in the urban slum environment. PLoS Negl. Trop. Dis..

[25] Kelly AH (2018). Shadowlands and darkcorners. Med. Anhropol..

[26] Keenlyside RA, McCormick JB, Webb PA, Smith E, Elliott L, Johnson KM (1983). Case-control study of Mastomys natalensis and humans in Lassa virus-infected households in Sierra Leone. Am. J. Trop. Med. Hyg..

[27] Olson DM, D. E., Wikramanayake ED, Burgess ND, Powell GVN, Underwood EC et al. Terrestrial Ecoregions of the World: A New Map of Life on Earth. A new global map of terrestrial ecoregions provides an innovative tool for conserving biodiversity. Bioscience. *Oxford University Press* **51**, 933–938 (2001).

[28] Fichet-Calvet E, Koulemou K, Koivogui L, Soropogui B, Sylla O, Lecompte E (2005). Spatial distribution of commensal rodents in regions with high and low Lassa fever prevalence in Guinea. Belg. J. Zool..

[29] Mills JN, C. J., Ksiazek TG, Peters CJ, Velleca WM. Methods for trapping and sampling small mammals for virologic testing *Centers for Diseases Control and Prevention* (1995).

[30] Fichet-Calvet, E. in *The role of animals in emerging viral disease* (ed Johnson N) 89–123 (Elseviers 2014).

[31] Team, R. C. *R: A language and Environment for Statistical Computing* <http://www.r-project.org> (2018).

[32] White GC (1996). Analysis of frequency count data using the negative binomial distribution. Wiley Ecol. Soc. Am..

[33] Ripley B, V. B., Bates DG, Hornik K, Gebhardt A, Firth D. MASS:Support Functions and Datasets for Venables and Ripley's MASS. (2002).

[34] QH, V. (1989). Likelihood ratio test for model selection and non-nested hypotheses. Econometrica.

[35] Jackman S, T. A., Zeileis A, Maimone C, FEaron J. pscl: Political Science Computational Laboratory (2017).

[36] Bolker BM, Clark CJ, Geange SW, Poulsen JR, Stevens MHH (2009). Generalized linear mixed models: A practical guide for ecology and evolution. Trends Ecol. Evol. Elsevier Curr. Trends.

[37] Bates D, Bolker B, Walker S. Ime4: Linear Mixed-Effects Models using 'Eigen' and S4. (2018).

[38] Greenland S (2015). Statistical fondations for model-based adjustments. Ann. Rev..

[39] Zuur AF, L. E., Walker NJ, Saveliev A, Graham M. S. (2009). Mixed effects models and extensions in ecology with R.

[40] Zuur AF, Elphick CS (2010). A protocol for data exploration to avoid common statistical problems. Methods Ecol. Evol..

