# Supplementary material for: Domestic risk factors for increased rodent abundance in a Lassa fever endemic region of rural Upper Guinea
Source: Sci Rep. 2021 Oct 19;11:20698. doi: 10.1038/s41598-021-00113-z (PMC8526584; doi:10.1038/s41598-021-00113-z)
Supplement: Supplementary file 1 — Supplementary Information 1. [file 41598_2021_113_MOESM1_ESM.docx]

Tables

**Table 1.** Distribution of demographic characteristics and rodent contact of participants.

| **Variable** | **Number (%) of participants** |
| --- | --- |
| **Age group** | |
| *<25* | 60 (14.8) |
| *25-49* | 229 (56.5) |
| *50-74* | 92 (22.7) |
| *75+* | 24 (5.9) |
| **Gender** | |
| *Female* | 288 (71.1) |
| *Male* | 117 (28.9) |
| **Ethnicity** | |
| *Malinke* | 240 (59.3) |
| *Djallonke* | 141 (34.8) |
| *Other* | 24 (5.9) |
| **Education level** | |
| *No formal education* | 269 (66.4) |
| *At least some formal education* | 136 (33.6) |
| **Occupation** | |
| *Agriculture* | 250 (61.7) |
| *Housewife* | 96 (23.7) |
| *Retired* | 20 (4.9) |
| *Other* | 39 (9.6) |
| **Contact with rodents** | |
| *Yes* | 301 (74.3) |
| *No* | 104 (25.7) |
| **Contact with rodent excrement** | |
| *Yes* | 300 (74.1) |
| *No* | 105 (25.9) |
| **Owns a cat** | |
| *Yes* | 46 (11.4) |
| *No* | 359 (88.6) |
| **Owns a dog** | |
| *Yes* | 66 (16.2) |
| *No* | 339 (83.7) |
