# Supplementary material for: Domestic risk factors for increased rodent abundance in a Lassa fever endemic region of rural Upper Guinea
Source: Sci Rep. 2021 Oct 19;11:20698. doi: 10.1038/s41598-021-00113-z (PMC8526584; doi:10.1038/s41598-021-00113-z)
Supplement: Supplementary file 2 — Supplementary Information 2. [file 41598_2021_113_MOESM2_ESM.docx]

**Table 2: Variance inflation factors for the fully adjusted negative binomial model**

| **Variable** | **Variance inflation factor** |
| --- | --- |
| Village | 2.64 |
| Building type | 2.85 |
| Floor material | 2.65 |
| Floor condition | 1.65 |
| Room purpose | 2.28 |
| Porosity level | 2.12 |
| Burrows present | 1.83 |
| Food present | 1.05 |
| Water present | 1.67 |
| Garbage storage location | 1.64 |
| Exterior grass present | 1.43 |
| Season | 1.41 |
